# Cefepime-induced Neurotoxicity: Five Cases Reported in a Single Institution

**DOI:** 10.7759/cureus.3666

**Published:** 2018-11-30

**Authors:** Kevin Schlidt, Andrew Kadlec, Sanjay Bhandari, Pinky Jha

**Affiliations:** 1 Student, Medical College of Wisconsin, Wauwatosa, USA; 2 Medicine, Medical College of Wisconsin, Milwaukee, USA; 3 Medicine, Medical College of Wisconsin, Brookfield, USA

**Keywords:** cefepime, encephalopathy, neurotoxicity, renal failure

## Abstract

Cefepime is a fourth-generation cephalosporin widely used to treat gram-positive and gram-negative infections. Its half-life is approximately two hours in patients with normal renal function and may increase to 13.5 hours in patients with acutely or chronically impaired renal function. Although dosage adjustment is recommended for patients with renal insufficiency to prevent drug accumulation, toxicity has been reported in patients even with normal renal function. One underreported complication of cefepime toxicity is cefepime-induced encephalopathy (CIE). While the pathophysiology is unclear, treatment involves early discontinuation of this antibiotic to decrease morbidity and mortality. We report five cases of cefepime-induced encephalopathy occurring within one year at a single institution.

## Introduction

Cefepime, a fourth-generation cephalosporin, has an antibacterial spectrum that covers aerobic gram-positive and gram-negative bacteria, including pseudomonas [1-2]. It is recommended for a wide array of infections including hospital-acquired pneumonia, febrile neutropenia, skin/soft tissue infections, and intra-abdominal infections [1-3]. Like other cephalosporins, cefepime is primarily excreted unchanged in the urine; the half-life is two hours with normal renal function, but can increase up to 13.5 hours in the presence of impaired renal function. To avoid adverse drug events, dosage adjustments are recommended in the setting of renal insufficiency [4]. In 2002, the Food and Drug Administration (FDA) adjusted the labeling to account for an increased risk of seizures, encephalopathy, and myoclonus, particularly in the setting of renal impairment [3]. These adverse events were mainly seen in patients 65 years old or greater with renal dysfunction but without proper dose adjustments, with an incidence of 56%. Improved and early recognition may help to decrease morbidity and mortality, and generate a more accurate estimate of true incidence. The current case-series includes five cases of cefepime-induced encephalopathy (CIE) diagnosed within one year at a single tertiary-care academic center, with a resolution of symptoms following discontinuation of cefepime.

## Case presentation

Case 1

A 63-year-old man with a history of type 2 diabetes mellitus complicated by a prior stroke, chronic foot ulcers, and end-stage-renal disease (ESRD) on hemodialysis presented with a fever and increased drainage from a right foot ulcer. A computed tomography (CT) scan of his foot showed cortical destruction and sclerosis consistent with osteomyelitis. The patient underwent a toe amputation and a six-week course of intravenous (IV) cefepime 1g every 24 hours and vancomycin 1,750mg with hemodialysis three days a week. Three days after starting cefepime, the patient became confused during hemodialysis and had difficulty grasping objects with his right hand. The head CT was negative for acute intracranial pathology and his laboratory tests were unremarkable. Brain magnetic resonance imaging (MRI), lumbar puncture, and electroencephalogram (EEG) did not reveal the cause of his encephalopathy. Despite the cessation of all sedating and psychotropic medications, the mental status failed to improve. Review of the patient’s medical records showed that he had received cefepime, with dosing unadjusted for his impaired renal function, for two days following his procedure. Cefepime was promptly discontinued which corresponded to the 12th day of hospitalization. He was then started on ertapenem. His mental status returned to baseline two days later without any neurological sequelae. He continued to be on ertapenem along with vancomycin without manifesting any further encephalopathy during the remaining part of his hospital course.

Case 2

A 65-year-old female with a past medical history of lupus, hypertension, ESRD on dialysis, and recent left lower extremity graft repair presented to the hospital with complaints of fever, pain, and redness around her graft site. Upon admission, she was febrile and tachycardic with a white blood cell count of 30,000/cubic millimeter. Physical examination revealed erythema and tenderness around her left lower extremity graft site. Transplant surgery was consulted for debridement of her infected graft site, and she was started on IV vancomycin and cefepime 2g every 24 hours. On the second day of hospitalization, she underwent surgery but experienced right arm weakness, left eye deviation, and aphasia postoperatively. The patient was transferred to the neuro ICU where she required intubation for airway protection. The CT angiogram and brain MRI were both negative, and the electroencephalogram (EEG) showed diffuse triphasic waves and severe generalized slowing. Given the unremarkable workup, the infectious disease service recommended holding cefepime, which resulted in an improvement of mental status two days after stopping the drug. The patient was extubated and transferred to the medicine floor in stable condition.

Case 3

A 70-year-old African-American female with a past medical history significant for non-ischemic cardiomyopathy, pulmonary hypertension, chronic kidney disease (CKD) stage III, and ankle fracture status post open reduction and internal fixation complicated by a wound infection, presented to our hospital with word-finding difficulty. Three weeks prior, the patient was hospitalized for a wound infection of her surgical site with wound cultures positive for pseudomonas and enterococcus. She subsequently underwent surgical debridement, incision and drainage, and was started on IV piperacillin/tazobactam 4.6g every six hours. She was later discharged to a subacute rehabilitation on IV vancomycin 1,250mg every 24 hours and cefepime IV 2g every 12 hours. At the rehabilitation facility, the patient’s daughter noted that the patient’s cognitive ability had continued to deteriorate since discharge from the hospital. The patient now had word-finding difficulty prompting an emergency department (ED) evaluation for a stroke. The vitals in the ED were within normal limits and the physical exam only remarkable for asterixis. Complete blood count showed anemia and mild leukocytosis and basal metabolic panel was remarkable for a blood urea nitrogen of 38mg/dL (ref range: 7-20mg/dL), bicarbonate of 19mEq/L (ref range: 23-29mEq/L), and a creatinine of 4.66mg/dL (ref range: 0.8-1.4mg/dL). CT, chest x-ray, ultrasound, and MRI did not identify any acute processes contributing to her presentation. An EEG suggested moderate diffuse cerebral dysfunction (encephalopathy) with possible structural or physiologic disturbances in the left hemisphere. Due to the high dose of antibiotic in the setting of chronic renal disease, there was a high suspicion for cefepime-induced neurotoxicity. Infectious disease switched the antibiotic regimen to IV meropenem 1g every 24 hours, and the patient experienced a drastic improvement in mentation. The patient was subsequently discharged back to subacute rehabilitation to finish her antibiotic course for wound infection.

Case 4

A 63-year-old Caucasian woman with a past medical history of type 2 diabetes, neurogenic bladder, and a recent diagnosis of bilateral hydronephrosis was re-admitted due to worsening weakness and confusion. Two weeks prior to her re-admission, she had presented to an outside hospital for abdominal cramping and was found to have an obstructive urinary tract infectioin (UTI) with growth of candida glabrata on urinalysis. Urology was consulted for her complicated pyelonephritis with hydronephrosis, ultimately leading to bilateral stent placement. At this time, she had a creatinine of 1.2mg/dL. The patient was then discharged on fluconazole 200mg every 12 hours and cefepime 2g every 12 hours empirically for two weeks. One week later, she had complaints of weakness, difficultly ambulating, and confusion. At baseline, the patient was functional and alert and oriented to time, place, and person. However, upon admission, she was confused and oriented to only name and place but could not recall the name of the hospital. Vitals were unremarkable except for mild tachycardia (110/minute). Physical examination was significant for suprapubic region tenderness upon palpation. The repeat urinalysis was positive with culture growing candida glabrata. Head CT and other laboratory results were unremarkable. She was started on IV hydration and continued on cefepime and fluconazole. Urology performed a CT cystogram, which showed findings consistent with a combination of cystitis and partial disruption of the bladder dome, and the patient was subsequently continued on her Foley catheter that was started during the admission. Antibiotics were discontinued after the patient completed the two-week course. Within 24 hours of cessation of the antibiotics, the patient’s mental status improved. Due to persistent suprapubic pain along with re-growth of candida in the urine culture, the patient was restarted on fluconazole, which was subsequently changed to amphotericin deoxycholate for seven days based on sensitivities and infectious disease’s recommendations. The patient’s mental status returned to baseline during hospitalization in parallel with an improvement in her creatinine and discontinuation of cefepime, and the patient was discharged to a subacute rehabilitation facility.

Case 5

A 60-year-old male with a past medical history of asthma, spastic paraplegia, hypertension, hyperlipidemia, peptic ulcer disease, and tibial osteomyelitis post-infected hardware removal was admitted for altered mental status. He was admitted to the orthopedic service for infected hardware removal one month prior to the current admission. He was subsequently started on IV cefepime 2g every eight hours for tibial osteomyelitis and then discharged to a skilled nursing facility for six weeks. Approximately 17 days later, the staff at the nursing facility reported that the patient was delirious, slurring his speech, and pulling out his peripherally inserted central catheter (PICC) line in the night. He was then hospitalized at an outside facility for four days where he had an extensive workup, including a CT head, MRI head, and EEG without any conclusive etiology for his altered mental status. The EEG showed generalized slowing and evidence of metabolic encephalopathy and he was discharged. He returned to an outside emergency department three days later for persistent neurological symptoms, where he was found to have acute kidney injury. Following administration of IV fluids, he was discharged to his skilled nursing facility. His mental status did not improve. After consultation with the infectious disease team, the patient was admitted to our hospital for further workup. Upon admission, his vitals were unremarkable and physical examination showed confusion and disorientation without any other focal neurological deficits. Laboratory results were unremarkable except for an elevated creatinine of 1.8mg/dL (baseline 1-1.2mg/dL) indicative of unresolved acute kidney injury. Cefepime was discontinued, and the patient’s mental status and speech improved over the next 72 hours. Nephrology was consulted. After extensive workup, acute kidney injury was presumed to be secondary to cefepime toxicity with a component of acute tubular necrosis given the hyaline and granular casts seen on urinalysis. The patient was later discharged back to the facility where he had no further episodes of confusion or altered mental status.

## Discussion

The five patients discussed here demonstrated a consistent presentation of an acute change in mental status while receiving cefepime, a thorough but quite unremarkable neurological workup, and improvement in symptoms upon cessation of cefepime. Cases one, two, and three had underlying renal disease, while cases four and five had a normal baseline renal function prior to the initiation of cefepime. Cefepime-induced encephalopathy is a rare and under-reported diagnosis.

Cefepime is a parenteral fourth-generation antibiotic widely used to treat infections. Compared to third-generation cephalosporins such as ceftazidime, cefepime use is associated with a lower incidence of anaphylaxis, seizures, neutropenia, and bleeding episodes [5]. The exact incidence of cefepime-induced encephalopathy is unknown, as it is likely under-recognized and under-reported. At our institution, there were five cases in a single year. Because cefepime is primarily (85%) excreted by the kidneys, drug accumulation can occur in patients with impaired renal function. While cefepime-induced neurotoxicity, presenting as encephalopathy, myoclonus, seizures, non-convulsive status epilepticus, and mortality, is more common if renal dysfunction is present, it can also occur with normal renal function [4]. Predisposing factors for neurotoxicity include a high cefepime dose (>4g/day), reduced renal drug clearance, increased unbound antibiotic levels, and increased drug cerebral penetration [2]. In patients with renal disease, the maintenance dose should be reduced, and the patient closely monitored for neurotoxicity. Symptoms usually present between one and ten days after administration of cefepime and resolve within two to seven days following discontinuation. If unrecognized, cefepime-induced encephalopathy can have severe, sometimes fatal outcomes [4]. The exact mechanism of the neurotoxicity is unknown, although some studies have suggested that beta-lactam antibiotics competitively inhibit gamma-amino butyric acid (GABA)-induced chloride currents via direct binding and subsequently inhibit the inhibitory response, which causes depolarization of the postsynaptic membrane potential and contributes to neurological symptoms, including seizure [2].

A study by Jallon et al. in 2000 reported 19 cases of encephalopathy over a three year period associated with cefepime usage [6]. The patients’ ages ranged from 57 to 91 years and all had underlying renal insufficiency. Each case presented with confusion and EEGs displaying diffuse, rhythmic triphasic sharp waves. Following discontinuation of cefepime, clinical symptoms resolved. Another study in 2005 examined three cases with cephalosporin-induced non-convulsive status epilepticus in the setting of underlying renal insufficiency, with each case presenting altered mental status associated with epileptiform activity of continuous or almost continuous rhythmic generalized bi-triphasic sharp waves on electroencephalography [7].

Garces et al. conducted a prospective cohort study that followed 498 patients using cefepime between February 2005 through February 2006 [8]. When cefepime was suspected as the probable cause of encephalopathy with all other metabolic problems ruled out, EEGs were performed to assist in the diagnosis, with the first performed during cefepime use and another performed at least 48 hours following drug discontinuation and/or clinical improvement [9]. Five patients in the study were diagnosed with cefepime-induced encephalopathy, indicating a cumulative incidence of approximately 1% (0.01). In this cohort, 111 patients had a mean glomerular filtration rate (GFR) <60 ml/min; mean GFR in patients with encephalopathy (n=5) was 17.20ml/min + 10.75ml/min and, in patients without encephalopathy (n=106), mean GFR was 32.59ml/min + 14.89ml/min. This study suggested that the development of cefepime-induced encephalopathy may be related to the severity of impairment in glomerular function [8], which was consistent with the findings from our institution. Three out of five of our patients had underlying renal disease, and the other two cases presenting with CIE had concurrent acute kidney injury.

Payne et al. performed a systematic review which included 135 cases of CIE [8]. The cohort median age was 69 years old. Majority of the patients presented with renal dysfunction (80%) and required intensive care (81%). All patients exhibited altered mental status, with other symptoms including reduced consciousness (47%), myoclonus (42%), and confusion (42%). Ninety-eight of the 135 patients (73% of the cohort) had EEGs with abnormalities, including triphasic waves (40%), focal sharp waves (39%), non-convulsive epilepticus (25%), and myoclonic status epilepticus (7%). Per drug dosing guidelines, it was determined that 48% of patients were overdosed with 26 experiencing neurotoxicity despite appropriate dosing. The mean cefepime serum and CSF concentrations were 56mg/L (n=21) and 13mg/L (n=4), respectively. Symptoms improved in 89% of patients, with 87% surviving. Symptoms began, on average, four days following drug initiation and resolution occurred, on average, two days following discontinuation of cefepime, antiepileptic administration or hemodialysis interventions, consistent with the time-to-onset and time-to-resolution observed in the above cases [10].

Three meta-analyses measured the all-cause mortality of cefepime with conflicting results [10]. The first study in 2006 with 33 trials evaluated antibiotic treatment for neutropenic fever, finding a higher all-cause mortality after 30 days with cefepime when compared to other beta-lactam antibiotics (RR 1.04, 95% CI 1.24-3.07) [3, 11]. Yahav et al. in 2007 performed a meta-analysis with 57 randomized trials comparing cefepime with other beta-lactam alternatives and found a higher all-cause mortality as well. This study suggested that the causes may be from unrecognized adverse effects such as encephalopathy [2-3]. In contrast, in 2008, the FDA performed a meta-analysis with 88 clinical trials including the trials from Yahav et al. and reported no significant difference in observed mortality [3, 12].

Cefepime has been shown to have significant adverse effects, even with renal dose adjustments. In 2013, Fugate et al. evaluated 100 patients for neurotoxicity in the ICU setting and found 15 patients who likely had cefepime encephalopathy and 7 patients who definitely had cefepime encephalopathy [13-14]. This study found that four of the patients had cefepime doses adjusted for renal function, but still experienced adverse effects [2], suggesting neurotoxic effects despite normal renal function. In 2005, a case with a 79-year-old female with normal function became actively confused while being treated with cefepime for a pseudomonal urinary tract infection [14]. Her EEG showed generalized sharp and slow wave discharges. The patient’s mental status did not change with treatment with lorazepam and valproic acid but returned back to baseline over three days once cefepime was discontinued [15]. Another case report in 2005 discussed cefepime and cefixime-induced encephalopathy in a patient with normal renal function whose acute delirium resolved following discontinuation of the cephalosporins on two separate occasions [2, 16]. A study in 2011 showed epileptiform discharges in 14 of 1120 patients receiving cefepime, most of whom had normal renal function [2, 17]. A recent case report by Andrew Meiller et al. reported an additional case of cefepime-induced encephalopathy in a patient with normal renal function; a 75-year-old male being treated with cefepime for pneumonia developed altered mental status that improved three days following discontinuation of cefepime with no other explanation, despite an extensive workup [2].

Our study found cefepime-induced neurotoxicity in the setting of acute or chronic renal dysfunction. As with other studies, other causes of encephalopathy were first ruled out, including infectious, metabolic, and neurologic etiologies. Like other studies, the EEG had diffuse slowing with triphasic waves [2]. However, only two of the five EEGs were positive for the characteristic EEG changes normally associated with CIE. While EEGs may be beneficial to order to better determine the etiology of the affected mental function, the absence of the characteristic cefepime-induced EEG findings does not appear to rule out the possibility of CIE.

The cases presented here further illustrate how cefepime-induced encephalopathy is a diagnosis of exclusion, requiring an extensive workup and early recognition. Notably, despite an incidence of approximately 3% in other studies [18], we identified five cases in a single year at a single institution, suggesting that the true incidence is likely much higher than previous estimates. The discrepancy likely results from lack of awareness of this drug-related adverse effect and thus underdiagnosis. Therefore, we suggest that clinicians should increasingly consider the diagnosis of CIE in cefepime-treated patients presenting with altered mental status who present with a generally unremarkable workup for common causes of acute neurological changes, along with suggestive EEG findings.

## Conclusions

Cefepime-induced neurotoxicity is a challenging diagnosis. Clinicians should maintain a high index of suspicion of drug-induced encephalopathy in all patients presenting with altered mental status during the course of cefepime treatment, especially in the absence of other identifiable sources. Further research is needed to identify the mechanism of cefepime-induced encephalopathy, additional risk factors, and true incidence. While there are relatively few cases of cefepime-induced neurotoxicity reported, the presence of five known cases at our institution within a one-year timespan suggests that this condition is likely vastly under-reported and under-diagnosed.
